# The impact of paternal age on new mutations and disease in the next generation

**DOI:** 10.1016/j.fertnstert.2022.10.017

**Published:** 2022-12

**Authors:** Katherine A. Wood, Anne Goriely

**Affiliations:** a Radcliffe Department of Medicine, MRC Weatherall Institute of Molecular Medicine, University of Oxford, Oxford, United Kingdom; b National Institute for Health and Care Research (NIHR) Oxford Biomedical Research Centre, Oxford, United Kingdom

**Keywords:** Paternal age effect, selfish selection, spermatogonial stem cells, rare disorders, complex disorders

## Abstract

Advanced paternal age is associated with an increased risk of fathering children with genetic disorders and other adverse reproductive consequences. However, the mechanisms underlying this phenomenon remain largely unexplored. In this review, we focus on the impact of paternal age on de novo mutations that are an important contributor to genetic disease and can be studied both indirectly through large-scale sequencing studies and directly in the tissue in which they predominantly arise—the aging testis. We discuss the recent data that have helped establish the origins and frequency of de novo mutations, and highlight experimental evidence about the close link between new mutations, parental age, and genetic disease. We then focus on a small group of rare genetic conditions, the so-called “paternal age effect” disorders that show a strong association between paternal age and disease prevalence, and discuss the underlying mechanism (“selfish selection”) and implications of this process in more detail. More broadly, understanding the causes and consequences of paternal age on genetic risk has important implications both for individual couples and for public health advice given that the average age of fatherhood is steadily increasing in many developed nations.

**DIALOG:** You can discuss this article with its authors and other readers at https://www.fertstertdialog.com/posts/35819

It has long been known that older parents have a high risk of having children with genetic disorders. The link between advanced maternal age and congenital abnormalities, particularly those associated with chromosomal aneuploidies, in offspring has received considerable attention, e.g., the strong association between maternal age effect and Down syndrome (trisomy 21) prevalence (1). However, there is a growing body of evidence indicating that independent of the maternal age, elevated paternal age is associated with difficulties conceiving, complications in pregnancy, an increased susceptibility of offspring to a wide range of conditions including spontaneous dominant disorders, congenital abnormalities, neurodevelopmental conditions, and various malignancies (2, 3, 4, 5, 6, 7, 8, 9, 10, 11, 12). The *American Society* *for* *Reproductive Medicine*, the *British Andrology Society,* and the *Canadian Fertility and Andrology Society* have advised that the upper limit for sperm donors for assisted conception should be 40 years old as a precautionary measure “so that the potential hazards related to aging are diminished” on the basis of increased risk of genetic abnormalities in children (13, 14, 15).

In many developed countries, the average age of fatherhood has been steadily increasing, despite considerable demographic variations. In the United Kingdom, e.g., the standardized mean age of fathers in 2020 was 33.7 years, the highest since data collection began and an increase from 29.7 years in 1970, whereas over the same period mean maternal age rose from 26.7 to 30.7 years (16). A similar picture is apparent in the United States, with one study indicating that mean paternal age has risen from 27.4 years in 1972 to 30.9 years in 2015 (with variation attributed to ethnicity or race, geographic location, and education level), with 8.9% and 0.9% of fathers over the age of 40 and 50, respectively (17). Given this trend prevalent across the developed world, there is an ever-increasing need to evaluate the impact, and understand the causes and consequences, of advanced paternal age on genetic risk for both individual couples and public health advice. Moreover, this information is also crucial given the popularity of assisted reproductive technologies that offer couples the option to reproduce later in life, to provide accurate risks regarding delayed parenthood (18).

Although epidemiological studies have shown a convincing correlation between paternal age and disease risk, in many cases, this association is not well defined and (in some cases) not always reproducible (8, 19, 20, 21). Often, the exact threshold of what consists of an “advanced” paternal age is also poorly defined and varies from study to study (9). Additional factors can further cloud our interpretation of population-based studies, including the fact that maternal and paternal ages are often highly correlated with little variability between the age of the mother and the father, so unpicking the impact of one from the other in terms of disease association can be challenging. Importantly, correlations do not provide direct evidence for causality, and the mechanisms underlying the effect of advanced paternal age on disease remain uncertain and are likely to be moderated by a complex interaction of factors (12).

Over the last decade, thanks to the advances and falling costs of next generation sequencing technology, it has become possible to interrogate and further dissect the components mediating the effect of paternal age on disease risk. Here, we focus largely on de novo point mutations (DNMs), DNA sequence variations that are “new” in offspring and are not apparently present in either parent. In this review, we examine how, why, where, and how often new mutations are introduced in our genomes, and the link between DNMs, disease, and other negative reproductive outcomes. We then discuss the unusual properties of a small group of specific genetic disorders (“paternal age effect” (PAE) disorders) that have provided a paradigm to study DNMs directly in their tissue of origin and led to the discovery of the process of “selfish selection.” These DNMs are important contributors to human disease, and understanding their origins and the factors that influence their occurrence, such as advanced paternal age, have important implications for public health, assisted reproductive technology treatments, complex disease, and the evolution of our genome.

## Origins and frequency of new mutations in humans

**Figure 1:**
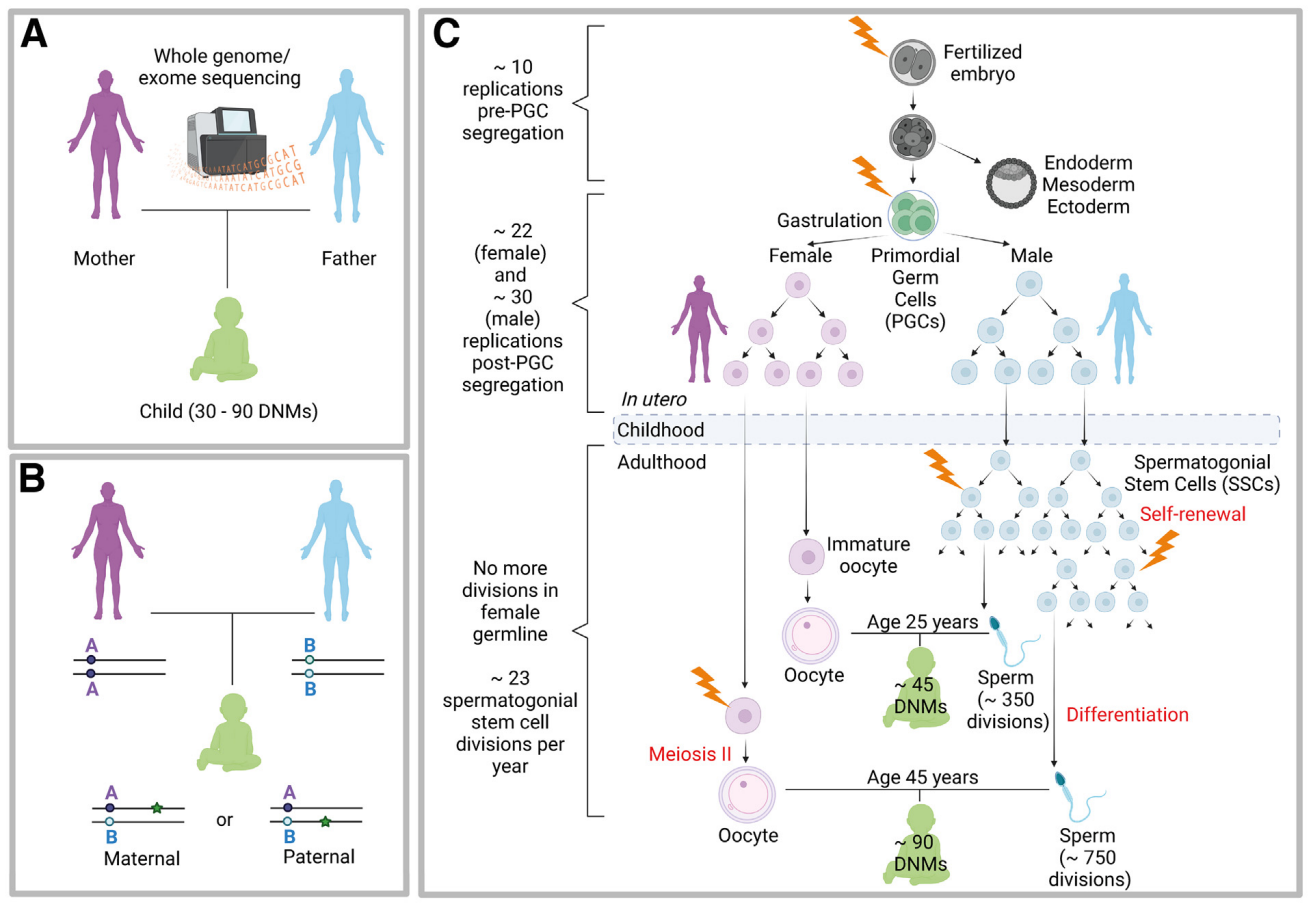
Origins of de novo mutations: **(A)** Sequencing of the whole genome or whole exome of both biological parents and a child (trio sequencing) allows identification of new mutations only present in the child. Such studies have shown that each newborn acquires ∼30–90 de novo mutations (DNMs), depending on parental age at conception. **(B)** Determination of the parental origin of a DNM by haplotype phasing. When an informative heterozygous SNP - for example, the SNP is AA (purple) in the mother and BB (blue) in the father - is present in the vicinity of a DNM (green star) in the child, it can be used to distinguish the maternally- and paternally-derived alleles and determine the parent of origin of the DNM. **(C)** Gametogenesis and origins of DNMs. In humans, segregation of PGCs from somatic lineages occurs after ∼10 mitoses, just before gastrulation takes place. Embryonic germ cells then undergo a few more replications (∼22 in females and ∼30 in males). After birth, oocytes do not undergo any further mitotic divisions. However, throughout adulthood, spermatogonial stem cells SSCs actively replicate to sustain sperm production, dividing every ∼16 days (∼23 divisions per year). It can be estimated that the sperm produced from a 25-year old male has undergone ∼350 replications, while ∼750 SSC replications would have taken place to sustain sperm production in a 45-year old male. These differences in germ cell biology likely account for the observed 80:20 ratio of paternal to maternal DNMs observed in offspring, the majority of which arise from copying errors during SSC cycling, with the number of DNMs doubling with every additional 20 years of paternal age. Lightning bold represents a mutational event. Figures created with BioRender.com. PGC = primordial germ cell; SNP = single nucleotide polymorphism; SSC = spermatogonial stem cell.

The rate at which new mutations arise is crucial to our understanding of both genetic disease and genome biology. Much insight into the biology of DNMs has been gained from large-scale implementation of whole-genome sequencing (WGS) or whole-exome sequencing (WES) of mother-father-child family trios—sequencing of coding portions (WES) or the whole genomes (WGS) of a child and both biologic parents (Fig. 1A). Such studies have convincingly shown that the number of new point mutations present in a new born is on an average 60 (approximately 30–90, depending on parental age at conception), placing the average human germline mutation rate at approximately 1.2 × 10^-8^ per nucleotide per generation (22, 23, 24, 25, 26, 27). Overall, the number of DNMs increases steadily and relatively monotonically with parental age. It is also possible to determine the parental origin of DNMs using a haplotype phasing strategy. This can be performed directly using the WGS data from the family trio when an informative heterozygous single nucleotide polymorphism (SNP) is present in the vicinity of the DNM that allows the maternally and paternally-derived alleles in the child to be distinguished (Fig. 1B) (28, 29). Such phasing methods have shown that approximately 80% of all DNMs are present on the paternally-derived allele, and the number of DNMs in a child is predominantly influenced by the age of the father at conception (Fig. 1C) (23, 24, 25, 28).

On average, approximately 1–2 additional DNMs arise in the genome of a child per additional year in the age of the father (22, 23, 24, 30). Juxtaposed to this, a smaller (but significant) maternal age effect has been reported (23, 25, 26, 31) (Table 1). Aside from DNM datasets derived from family trios, WGS studies of individual multi-sibling families with large age differences between the first and last child have again shown that the predominant factor determining the number of DNMs in a child is the paternal age, and overall only a modest variability in familial mutation rates has been reported (24, 26, 32, 33).

**Table 1:** Characteristics and effects of parental age on prevalence of different classes of DNMs.

Origin of mutational event	Characteristics	Rate of increase with parent’s age	No. of DNMs in child (with 20–25-year-old parent)	No. of DNMs in child (with 40–45-year-old parent)
DNM occurring in the paternal germline	Most of the DNMs (∼80%) across the genome are on the paternally-derived allele. Most paternal DNMs have a specific mutational pattern or signature, characteristic of processes involving stem cell cycling, and induction of copy errors (23, 24, 25, 26, 27)	1–2 DNMs per year of paternal age	Approximately 35 DNMs	Approximately 70 DNMs
DNM occurring in the maternal germline	Approximately 20% of DNMs are found on the maternally derived allele. These often exhibit a distinct mutational signatures and are found as “clustered DNMs” in specific regions of the genome—characteristic of accumulation of double-strand break-induced mutations throughout oocyte aging (23, 25, 28, 31)	Approximately 1 DNM every 4 y of maternal age	Approximately 5 DNMs	Approximately 10 DNMs
Mosaic DNM: Mutational event occurring in the primordial germ cells of one of the parents, or in the offspring during early development (the first few mitotic divisions) of the fertilized embryo	DNMs caused by germline mosaicism in either one of the 2 parents are associated with an increased recurrence risk in future pregnancies. (28, 29, 30) Mosaic DNMs occurring during offspring’s early development can be difficult to distinguish from constitutive (heterozygous) DNMs (26, 30).	No change with parental age No bias in parental origin	Approximately 5–10 DNMs	Approximately 5–10 DNMs
Selfish DNM: causing paternal age effect disorder	Small subset of recurrent functional/pathogenic DNMs in genes clustering in specific spermatogonial stem cell pathways, which present with a high apparent mutation rate. Selfish DNMs are exclusively found on the paternally-derived allele. Associated with increased paternal age (56).	Strong correlation between disease prevalence and paternal age: fathers are significantly older than population average (2, 56). Exponential increase of the mutation levels in sperm with age of the donor (56)	DNA mutational events are rare, but once they occur, they lead to clonal expansion within the testis over time, because they encode functional mutant proteins with oncogenic-like properties, i.e., the relative increase with age depends on the activity and the selective advantage conferred by the mutant protein.	

DNA = deoxyribonucleic acid; DNM = de novo mutation; No. = number.

It has long been accepted that differences in the biology of the male and female germlines provide a compelling explanation as to why most (approximately 80%) of the DNMs are paternal in origin, in particular the number of germline cell divisions in the life history of a sperm compared with an egg (Fig. 1C) (Table 1) (34). All cell divisions take place during early embryogenesis that are required to produce an oocyte. By contrast, spermatogonial stem cells (SSCs) within the seminiferous tubules of the testis divide continuously to sustain sperm production throughout a man’s reproductive life, and so the number of genome replications increases with age. It can be estimated that the sperm produced by a 25-year-old man would have undergone approximately 350 SSC replications compared with approximately 750 in a 45-year-old man (Fig. 1C) (34). In addition to the number of mitotic divisions in the male germline resulting in incidental copying errors, other factors have been proposed that contribute to the age effect, including damage-associated mutations (particularly oxidative stress) during environmental exposures, age-related reduction in DNA repair and epigenetic reprogramming of germ cells (27, 35, 36, 37). However, molecular evidence derived from large WGS data is consistent with SSC replications being the predominant factor influencing the parental bias in DNM origin and the PAE of DNMs. For example, large WGS mutation datasets have been used to derive “mutational signatures” (defined as specific DNA substitution patterns typically caused by distinct underlying mutational processes, such as DNA replication errors, DNA damage caused by ultraviolet exposure, or other exogenous/endogenous exposure, defective DNA repair pathways) (38). This approach shows that the most common signatures observed in DNMs are similar to those associated with spontaneous preneoplastic somatic mutations (i.e., “mutation signatures 1 and 5”) (24, 25, 26, 39). This supports the idea that stem cell cycling is the main mutational process operative in the germline and the principal contributor to DNMs (24, 30, 38). Mutational signatures associated with paternally and maternally derived DNMs are distinct from one another, pointing that they originate through different processes (Table 1) (24, 25, 27, 30, 40, 41, 42).

More recently, in a large WGS study comparing mutational load across distinct histologic laser–microdissected tissues, Moore et al. (43) showed that the overall mutation rate (estimated 1.35 mutations per year of paternal age) and mutational signatures derived directly from the analysis of testicular stem cells was comparable to the mutation rate estimated from WGS trio studies (22, 23, 24). Therefore, these data provide further evidence consistent with copying errors within SSCs being the main source of DNMs (43).

However, the mutational rate of the germline is 1–2 orders of magnitude lower than in any other somatic cell types implying that other mechanisms maintaining genomic integrity must be at play (43, 44). Processes such as increased DNA repair capabilities or “transcriptional scanning” have been proposed to modulate the germline mutation signatures and mutation rate in human testes (27, 45). Xia et al. (45) proposed that widespread transcription in the testis (which expresses over 80% of protein-coding genes in humans) facilitates DNA repair and copy-error correction, thus reducing germline mutation rates (46, 47, 48).

## DNMs and genetic disease

Although DNMs are rare events, they are important contributors to genetic disease. In a seminal publication from the *United Kingdom Deciphering Developmental Disorders* study, a large-scale trio WES study of over 4000 families with severe, undiagnosed developmental disorders, the contribution of DNMs to spontaneous monogenic developmental disorders was estimated to be approximately 1 in 300 live births, greater than the combined impact of trisomies 13, 18, and 21 (49). Importantly, this study also showed a linear relationship between the prevalence of live births with dominant disorders caused by DNMs and parental age, doubling every 20 years (with prevalence estimated to range from approximately 1:448 [0.22% of all births] in young [20 years old] to approximately 1:213 [0.47%] in older [45 years old] couples), an almost identical slope to the relationship between paternal age and number of genome-wide characteristics of DNMs (49).

Overall, data on DNMs obtained from WGS/WES studies of large family trios and/or testicular tissues show that we all acquire a small but consistent number (approximately 30–90) of new mutations at each generation; there is a strong paternal bias in the origin of DNMs; most DNMs exhibit mutational signatures suggesting they occurred as copy errors during stem cell cycling; paternal age is the main contributor to the number of DNMs in a child, in most cases showing a linear relationship; and the prevalence of spontaneous developmental disorders shows a trend with paternal age almost identical to that observed for the genome-wide number of DNMs. Taken together, these findings strongly support the proposal that for most genetic disorders caused by DNMs, the effect of paternal age on disease prevalence is causally linked to the slow and steady accumulation of DNMs in SSCs over time.

There is robust epidemiological evidence that many other negative reproductive outcomes, such as preterm birth, low birth weight, poor Apgar scores, and increased morbidity, show a similar linear increase with paternal age; however, for these conditions, the link with DNM accumulation in SSCs is more tenuous (19, 50, 51, 52). For example, a study of over 40 million live births in the United States between 2007 and 2016 found a J-shaped association curve between paternal age and adverse perinatal outcomes after adjustment for maternal age, race, education, smoking status, and number of prenatal visits, with the youngest fathers having poorer reproductive outcomes than men in their 20s, that was followed by a steady and linear increase in negative pregnancy outcomes with increased paternal age (50). Given the linear relationship between paternal age and increased number of DNMs, it is tempting to suggest that many of the aforementioned adverse reproductive consequences are also caused by the DNM accumulation as a man ages; however, further evidence, including the role of genetic factors in these conditions, is required to support this hypothesis.

## Germline mosaicism, DNMs, and recurrence risk

Germline mosaicism is now recognized as another important source of DNMs and thus a genetic disease (Table 1). Spontaneous mutations can occur during early embryonic mitotic divisions in either one of the two parents—either before specification of the primordial germ cells resulting in mixed somatic and germline mosaicism or post-specification of the primordial germ cells, resulting in confined germline mosaicism. Moreover, these mutations may occasionally occur in the offspring post-fertilization (Fig. 1C) (29, 53). Although mosaicism has a small contribution to the overall DNM load, it has important clinical implications for sibling recurrence risk (29, 54). Because DNMs occurring spontaneously in the adult male or female germlines are rare events (mutation rate: approximately 1.2 × 10^-8^ per nucleotide/generation), the risk of the same DNM occurring as an independent mutational event is negligible. Conversely, in case of germline mosaicism, the DNM is present in multiple parental germ cells, leading to a substantial risk (up to 50%) of recurrence in future children (29). As a result, the average recurrence risk for conditions caused by DNMs is approximately 1–2% (24, 26, 29). A recent WGS study of paired blood–semen samples from 14 men showed that an average of approximately 30 mosaic variants were present (albeit most at very low levels) in their semen (55). The DNMs with a mosaic origin exhibit no parental age effect and no parental bias (the ratio of paternal to maternal mutations is approximately 50:50 compared with the approximate 80:20 ratio for germline DNMs).

## Selfish selection and PAE disorders

### PAE and Spontaneous Mendelian Disorders

When we consider the PAE in reference to genetic disease, there is a source of misunderstanding in the field which we seek to clarify. Although so far we have described the linear and remarkably similar relationship between DNMs, paternal age, and incidence of most developmental disorders, specific DNMs associated with a small subset of spontaneous Mendelian disorders show a nonlinear relationship with paternal age (therefore the birth prevalence of these disorders rises sharply as paternal age at conception increases) (2, 56). We previously defined these disorders as “PAE” disorders and proposed that additional mechanisms beyond a simple linear increase in DNMs across the genome arising from SSC replication underlies these conditions. The striking impact of advanced paternal age on these disorders has been recognized for over a century (2, 34). As such, because PAE disorders are well-characterized recurrent conditions with elevated birth prevalence that have received considerable attention, these are often considered to be exemplars for other spontaneous disorders. However, we must emphasize that PAE disorders are exceptions that differ from most of the spontaneous disorders already discussed in this review. Importantly the study of these rare disorders has provided novel insights into the intimate relationship that exists between the homeostatic regulation of SSCs and spermatogenesis, DNM prevalence and disease incidence.

The best known examples of PAE disorders are those caused by specific point mutations in *FGFR2* (causing Apert, Crouzon, and Pfeiffer syndromes); *FGFR3* (achondroplasia, thanatophoric dysplasia, hypochondroplasia, and Muenke syndrome); *RET* (multiple endocrine neoplasia types 2A and 2B); *PTPN11* (Noonan syndrome); and *HRAS* (Costello syndrome) (Table 2) (56). These disorders present with unusual features, including (56, 57):

• When the disorder is caused by a DNM, the causative mutations are (almost) exclusively inherited from unaffected fathers, pointing to the fact that the original mutational events occurred during spermatogenesis.
• A strong epidemiological PAE, whereby fathers of affected children are significantly older than the population average (approximately 2–7 years older than the population mean).
• A narrow mutational spectrum with the causative mutations encoding specific protein changes, typically associated with gain-of-function properties.
• A high apparent germline mutation rate, with individual substitutions occurring up to 1000 times more frequently than the average germline mutation rate.

**Table 2:** Paternal age effect genes in the RTK-RAS-MAPK pathway and associated Mendelian disorders.

Gene	Clinical disorder [OMIM reference number]	Reference
*FGFR2*	Apert syndrome [101200]	Goriely et al. (2003) (59)
	Crouzon syndrome [123500]	Goriely et al. (2005) (60)
	Pfeiffer syndrome [101600]	Qin et al. (2007) (61)
		Choi et al. (2008) (62)
		Yoon et al. (2009) (65)
		Maher et al. (2016) (71)
		Maher et al. (2018) (72)
*FGFR3*	Achondroplasia [100800]	Tiemann-Boege et al. (2002) (58)
	Thanatophoric dysplasia [187601]	Giudicelli et al. (2008) (63)
	Hypochondroplasia [146000]	Goriely et al. (2009) (64)
	Muenke syndrome [602849]	Shinde et al. (2013) (68)
		Maher et al. (2016) (71)
		Maher et al. (2018) (72)
*HRAS*	Costello syndrome [218040]	Giannoulatou et al. (2013) (67)
		Maher et al. (2016) (71)
*KRAS*	Cardiofaciocutaneous syndrome [615278]	Maher et al. (2016) (71)
	Noonan syndrome [609942]	Maher et al. (2018) (72)
*PTPN11*	Noonan syndrome [163950]	Yoon et al. (2013) (69)
		Eboreime et al. (2016) (70)
		Maher et al. (2016) (71)
		Maher et al. (2018) (72)
*RET*	Multiple endocrine neoplasia type 2A [171400]	Choi et al. (2012) (66)
	Multiple endocrine neoplasia type 2B [162300]	Maher et al. (2018) (72)
*BRAF*	Cardiofaciocutaneous syndrome [115150]	Maher et al. (2018) (72)
	LEOPARD syndrome [613707]	
	Noonan syndrome [613706]	
*CBL*	Noonan-like syndrome [613563]	Maher et al. (2018) (72)
*MAP2K1*	Cardiofaciocutaneous syndrome [615279]	Maher et al. (2018) (72)
*MAP2K2*	Cardiofaciocutaneous syndrome [615280]	Maher et al. (2018) (72)
*RAF1*	Noonan syndrome [611553]	Maher et al. (2018) (72)
	LEOPARD syndrome [611554]	
*SOS1*	Noonan syndrome [610733]	Maher et al. (2018) (72)

These features led to the hypothesis that the causative mutations should be present at elevated levels in testes and sperm and become more abundant in older men. Given the highly localized nature of the causative mutations (usually a specific point mutation), it was possible to test this hypothesis, although technically demanding, as the mutations are anticipated to be present at extremely low levels (ranging from approximately 10^-4^ to <10^-6^, based on the birth prevalence of the associated disorders). Nonetheless, specific pathogenic mutations in 12 genes (Table 2) that fulfill the above criteria were detected at elevated levels in sperm and/or testes and showed the anticipated PAE (58, 59, 60, 61, 62, 63, 64, 65, 66, 67, 68, 69, 70, 71, 72). Being able to quantify and visualize the mutations directly within the tissue in which they originate (sperm and testis) has allowed to define the mechanism by which these DNMs appear so frequently in the population, termed “selfish selection.”

**Figure 2:**
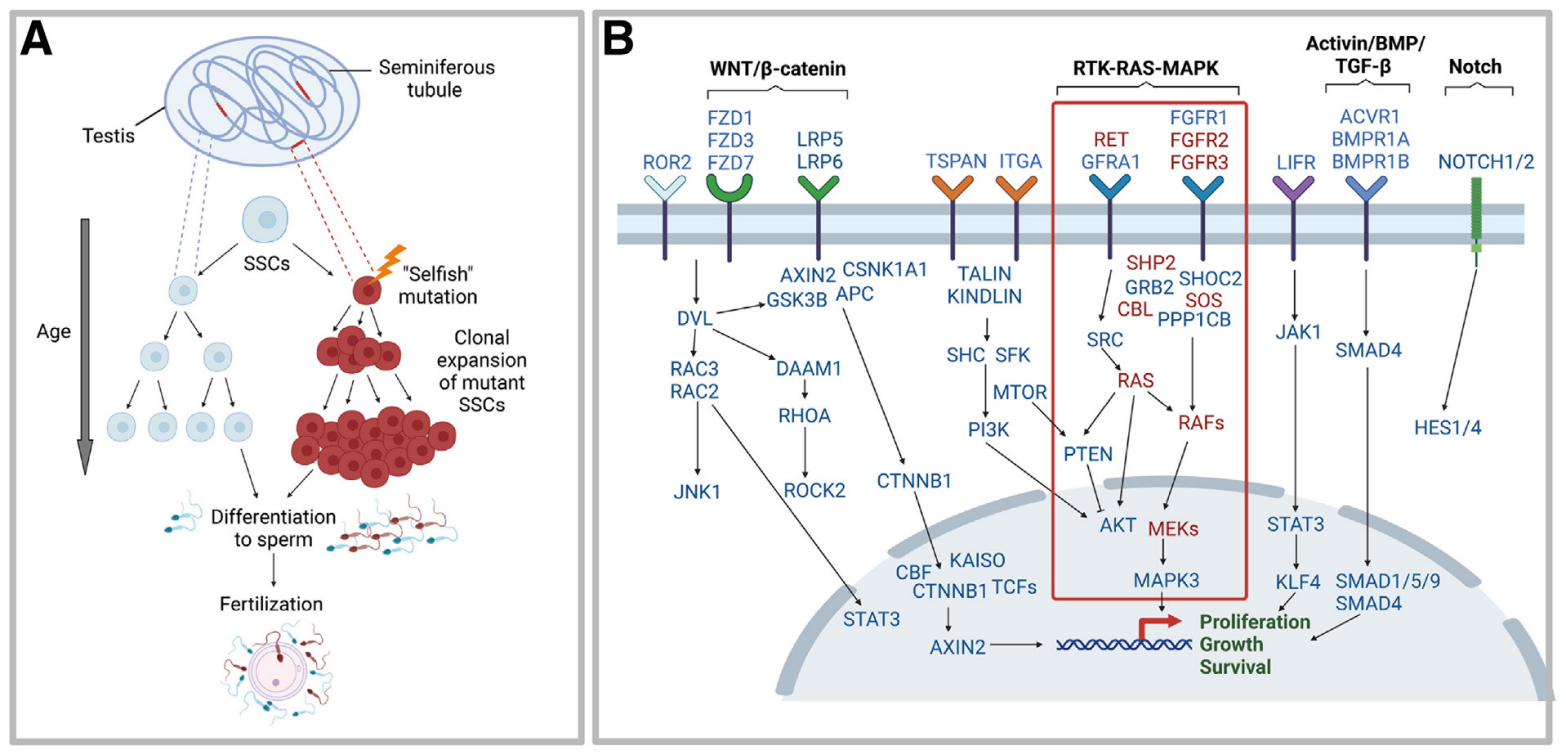
Selfish spermatogonial selection **(A)** In selfish selection, rare specific mutations occur in genes involved in the homeostatic regulation of spermatogonial stem cells (SSCs), conferring gain-of-function properties to the encoded protein. This provides the SSCs with a selective advantage over the wild-type neighbors and results in their clonal expansion within individual seminiferous tubules. As a consequence of clonal growth, the relative proportion of mutant sperm increases over the course of time. Fertilization of an oocyte by a sperm carrying a selfish mutation results in a genetic disorder in the offspring. This process is akin to tumorigenesis but occurs in the germline with consequences for the next generation. **(B)** Single-cell transcriptomics has allowed the key signaling pathways active in SSCs to be identified. To date, all known selfishly selected genes (highlighted in red) cluster within the Receptor Tyrosine Kinase (RTK)-RAS-MAPK pathway (red box). Deciphering the role of these pathways/genes in controlling proliferation, growth and survival of SSCs allows us to focus on new promising candidates for selfishly selected genes within the testes. Note that most of these genes cause genetic disease when mutated. Adapted from refs 74-75. Figures created with BioRender.com. SSC = spermatogonial stem cell.

In selfish selection, rare, specific point mutations that confer functional properties to the encoded protein spontaneously occur in SSCs within a seminiferous tubule of the adult testis, providing a competitive advantage and leading to clonal expansion of the mutant SSCs as the man ages (59, 60, 62, 64, 66, 67, 68, 69, 71, 72). In turn, this leads to a sharp increase in the relative mutation abundance in sperm over time and an increased likelihood of fertilization by a mutant sperm, resulting in the Mendelian disorder in the offspring (Fig. 2A). This process is equivalent to clonal growth observed in tumorigenesis; however, it occurs in the germline rather than the somatic tissues. Hence, selfish mutations have consequences not only for the individual in which they occur, causing a rare benign testicular tumor (spermatocytic tumor), but also for the next generation (56, 64, 73).

All selfishly selected mutations known to date cluster within the Receptor Tyrosine Kinase (RTK)-RAS-MAPK signaling pathway, the most frequently mutated pathway in cancer and a known regulator of testicular homeostasis, and the mutations all encode dominant gain-of-function, activating the pathway (57, 72). Based on our current understanding of the mechanism, we predict that *any* gene or pathway expressed in SSCs that controls testicular homeostasis could be under selection in the testis, provided that the mutations are compatible with the sperm viability and allow an embryo to develop. The technical demands in identifying mutations present at ultra-low levels in the male germline has precluded large-scale discovery screens, although rapid technical advances are opening the door to evaluate the process at scale (72). Over the last few years, single cell RNA-sequencing of human testes has allowed unbiased identification of the key molecular pathways that control the SSC homeostasis and provide important starting points for the discovery of additional selfishly selected genes (Fig. 2B) (74, 75, 76, 77, 78).

## Advanced paternal age and complex disorders

Thus far, we have shown that for many monogenic disorders there is an intimate relationship between paternal age, DNMs, genetic disease, and homeostatic regulation of the male germline, which strongly suggests a biologic link between these factors. However, most of the genetic disease is not caused by DNMs in single genes. Rather, “complex” (or common) diseases such as diabetes, neurodevelopmental conditions including autism and schizophrenia, and multiple sclerosis, are caused by a combination of multiple genetic (inherited variations and possibly DNMs), epigenetic, environmental, and lifestyle factors. Overall, these multifactorial disorders are poorly characterized, but for some of them, the association with advanced paternal age is robust and reproducible. For example, older fathers are at a high risk of having children with schizophrenia or autism (3, 19, 20, 79, 80, 81, 82). There is considerable debate as to whether the link between advanced paternal age and these disorders is due to: inherited genetic factors influencing the health of fathers and timing of reproduction (inherited model); testes-driven DNMs that arise randomly as a consequence of aging (de novo model); environmental factors (such as oxidative stress); epigenetic factors that accumulate over time; or (more likely) a combination of the above (12, 50, 83, 84, 85, 86, 87, 88). Unraveling the contribution of these factors to the PAE in common disorders is challenging (and beyond the scope of this review), but before this can be attempted, a better understanding of the etiology of these “complex” disorders will be required (12, 89). For example, Yoon et al. (87) recently showed that stratification of autism into two subtypes (simplex/low-risk families with a single affected child *vs.* multiplex/high risk with recurrent family history), provides an efficient way to identify families with likely causative de novo events*,* where they contribute to autism risk in up to 70% of the low-risk families.

For those complex disorders where the de novo contribution has been established or is likely to be high (12, 85, 86, 87, 90), it may be possible to ask whether selfish selection is a contributory factor to the PAE. To assess this possibility, it is important to consider the functional consequence of a particular selfish mutation both for the testis (where it provides a competitive advantage to mutant SSCs) and on the fitness of the offspring who inherits the constitutive mutation. Although “strong” selfish mutations causing the PAE disorders discussed above provide a robust selective advantage to SSCs and become significantly enriched in the sperm of older men, they cause deleterious disease phenotypes and poor reproductive fitness in the offspring, such that the variants are rarely transmitted over multiple generations. Although “weaker” selfish mutations are enriched to a lesser extent in SSCs and sperm, these are anticipated to cause more subtle and milder disease phenotypes. Importantly, these milder mutations can become a source of heritable material across several generations and contribute to the mutational burden characteristics of complex disorders (12, 90, 91). Further supporting this scenario are several studies which have shown that mutations in genes are known to operate in selfish selection, such as the RTK-RAS pathway or more globally in SSCs regulation, have been implicated in the pathogenesis of neurodevelopmental disorders (92, 93). Drawing from what we have learned from the study of PAE disorders, the general principles of age-related DNM accumulation and selfish selection predict that “mild” functional and pathogenic DNMs in specific growth-controlling pathways will accumulate and/or synergize to eventually dysregulate specific pathways causing disease in future generations through a mechanism we termed as the “global anticipation” of mutation accumulation. The steady increase of age of paternity in most developed countries is anticipated to accelerate this process, with the possibility of disease phenotypes manifesting over fewer generations. Transmission of newly acquired, mildly deleterious variants across generations may provide a parsimonious explanation for the association between advanced grandparental age and neurodevelopmental disorders (80, 94). Further evidence will be required to clarify whether DNM accumulation and/or selfish selection provide plausible mechanistic links to the PAE observed in some subclasses of neurodevelopmental (and more broadly, complex) disorders, but these hypotheses will become amenable to scrutiny as our understanding of these common human diseases broaden.

## What are the wider implications of selfish selection?

There are several corollaries of selfish selection with potential medical implications that are worth considering. First, the recurrence risk for selfish DNMs caused by selection is lower than that for other spontaneous disorders because the mutations arise and clonally expand during adulthood. Despite positive selection in the testis, the levels of selfish mutations in sperm will be lower than those observed for cases of parental mosaicism (Table 1). This has implications for genetic counseling and should be reassuring for the affected families as the recurrence risk for PAE disorders is predicted to be <0.1% (29, 95).

Second, the existence of selfish clones in the testes of all aging men (71, 72) may have implications for procedures involving biopsies for testicular sperm extraction combined with intracytoplasmic sperm injection as a treatment for male infertility. As an intriguing example, in a previous study we analyzed a testis from a 90-year old man with severe atrophy caused by strangulation in an inguinal hernia, and identified very few seminiferous tubules containing germ cells, consistent with the clinical presentation. However, on analysis, we showed that the remaining SSCs all carried the clonal FGFR3 p.K650E mutation, a known selfishly selected mutation that causes the lethal disorder thanatophoric dysplasia in offspring (and is associated with bladder carcinoma as a somatic event), implicating an apparent survival advantage of mutant compared with wildtype germ cells in this diseased testis (64, 71). This anecdotal finding raises the possibility that localized testicular biopsies used for sperm retrieval in men with overall poor spermatogenesis may be more prone to carry a selfishly selected DNM. Caution may need to be observed with biopsies performed in older men, and depending on the likelihood of selecting such surviving clones, screening of these testicular biopsies for known selfish DNMs may be advisable before use in testicular sperm extraction combined with intracytoplasmic sperm injection.

## Conclusions and perspectives

Over recent years, concerns have been raised about the impact of advanced paternal age on reproductive outcomes; however, remarkably little has been understood about the underlying biology driving the PAE. Recent advances in next generation sequencing technologies have facilitated large-scale trio WES and WGS studies as well as the characterization of DNMs at scale and in an unbiased manner. Such studies have shed light on some novel features of the germline in which the predominant contributor to the number of DNMs transmitted to offspring is the age of the father. Although this effect is small (an additional approximately 1–2 DNMs per extra year of fatherhood), it has a significant impact on spontaneous disease. With DNMs contributing to approximately 1:300 live births for severe spontaneous developmental disorders, this amounts to approximately 500,000 births annually worldwide—a considerable disease burden that cannot be ignored. The mutational signature of these DNMs is consistent with the copying errors that arise during the turnover of SSCs. The strong correlation between paternal age, the observed number of DNMs, and the prevalence of monogenic developmental disorders (and other negative reproductive outcomes) compellingly suggests that the steady accumulation of DNMs in the male germline contributes most significantly to the PAE.

However, we have emphasized that the classic PAE disorders are outliers to the above model. The unusual features of these disorders have allowed us to investigate selfishly selected mutations directly within sperm and testes and propose a unified mechanism for clonal expansion of mutant SSCs. Selfish selection is a universal process occurring in the testes of all men as they age. To date, it has only been possible to demonstrate that a handful of mutations causing a few spontaneous dominant disorders within the RTK-RAS-MAPK pathway genes are selfishly selected, but the study of large cohorts and the rapid improvements in technology are paving the way to investigate this process on a much larger scale (96, 97). Moreover, we speculate that increased age of fatherhood in the general population may lead to global anticipation of mutation accumulation that extends beyond the few rare PAE disorders characterized so far, to include a contribution to some complex and common disorders.

These findings have provided helpful insights to the biologic mechanisms driving epidemiological observations; however, as it is often the case in science, they have also raised new questions which should open novel research avenues. A key question in the field, which remains largely unanswered, is how the male germline maintains low mutation rate, despite SSCs dividing over decades, a process which is inherently mutagenic. Although DNMs accumulate with increasing paternal age, it is important to note that this phenomenon occurs in the backdrop of extremely low mutation rates, several orders of magnitude lower than in any other somatic cell types analyzed. Perhaps what is remarkable is not the fact that we mutate, but how few DNMs arise in our genome at each generation. Another notable feature of the germline that distinguishes it from somatic cells, is its resilience and robustness to mutagenic factors. Rather reassuringly, several WGS studies have shown that treatments with potentially mutagenic chemotherapeutic agents and/or exposure to ionizing radiation do not result in an increased incidence of DNMs or congenital malformations in offspring (98, 99, 100, 101, 102, 103). Confirming this expectation, a recent survey of DNM load from WGS data from approximately 22,000 family trios highlighted the remarkable consistency and monotonic linear increase with parental age of the numbers of DNMs. Indeed, only 12 individuals (0.05%) behaved as outliers, exhibiting 2–7 times more de novo single nucleotide variants than expected (27). Finally, there is no evidence of increased DNM load or altered mutation spectrums in mice or humans born as a result of assisted reproductive technology compared with spontaneously conceived offspring (104, 105).

Overall, the individual disease risk to offspring owing to advanced paternal age remains small; however, its impact on population health is nonnegligible. Moreover, the consequences of raising the age of fatherhood may need to be considered over several generations. Because DNMs occur in every newborn, it will be difficult to circumvent the genetic risks associated with paternal age; however, it is possible to anticipate the consequences of this phenomenon, for example, by offering prenatal screening for PAE conditions to older couples—similar to the testing provided routinely for the conditions associated with advanced maternal age. Finally, it is important to acknowledge that a small amount of new functional variants in our genome may also be desirable as this ensures the introduction of beneficial alleles in our genomes (106) and promotes genetic heterogeneity and diversity among individuals; these are essential attributes that have likely contributed to make us adaptable and successful as a species.
